# Significant effects of biologic therapy on lipid profiles and insulin resistance in patients with rheumatoid arthritis

**DOI:** 10.1186/s13075-015-0559-8

**Published:** 2015-03-07

**Authors:** Der-Yuan Chen, Yi-Ming Chen, Tsu-Yi Hsieh, Chia-Wei Hsieh, Chi-Chen Lin, Joung-Liang Lan

**Affiliations:** Division of Allergy, Immunology and Rheumatology, Taichung Veterans General Hospital and Faculty of Medicine, National Yang Ming University, Taiwan Boulevard, Taichung, 40705 Taiwan; Institute of Biomedical Science, National Chung Hsing University, No.250, Guoguang Rd., South Dist., Taichung, 40227 Taiwan; Rong Hsing Research Center for Translational Medicine, National Chung Hsing University, No.250, Guoguang Rd., South Dist., Taichung, 40227 Taiwan; Institute of Microbiology and Immunology, Chung Shan Medical University, No.110, Sec.1, Jianguo N.Rd., Taichung, 40201 Taiwan; Division of Immunology and Rheumatology, China Medical University Hospital, No. 2, Yude Rd., North Dist., Taichung, 40404 Taiwan; College of Medicine, China Medical University, No. 2, Yude Rd., North Dist., Taichung, 40404 Taiwan

## Abstract

**Introduction:**

The goal of this study was to investigate (1) the associations of rheumatoid arthritis (RA)-related inflammation or rheumatoid factor/anti-cyclic citrullinated peptide (anti-CCP) positivity with lipid profiles and insulin resistance (IR), (2) the effects of biologic therapy on lipid profiles and IR, and (3) potential predictors for the presence of subclinical atherosclerosis.

**Methods:**

Serum levels of lipid profiles were determined by enzymatic methods in 32 adalimumab-treated patients, 16 etanercept-treated patients, 24 tocilizumab-treated patients, and 20 biologic-naïve patients. Atherogenic index, which corresponds to the ratio of total cholesterol to high-density lipoprotein cholesterol (HDL-C), was calculated. IR was measured by homeostasis model assessment. Pro-inflammatory cytokine levels were examined by enzyme-linked immunosorbent assay. Common carotid artery intima-media thickness was determined by using sonography.

**Results:**

There was an inverse correlation between disease activity (disease activity score for 28 joints, or DAS28) and low-density lipoprotein cholesterol (LDL-C) levels (r = −0.226, *P* <0.05) and a positive correlation between DAS28 and IR (r = 0.361, *P* <0.005). Anti-CCP-positive patients had significantly higher DAS28 and IR compared with anti-CCP-negative patients. There was also a positive correlation between IR and levels of interleukin-6 or tumor necrosis factor-alpha (TNF-α). HDL-C levels significantly increased in patients receiving 6-month anti-TNF-α therapy, and levels of total cholesterol, LDL-C, and triglyceride increased in tocilizumab-treated patients. IR significantly decreased in patients under biologic therapy but was unchanged in biologic-naïve patients. Age, IR, and DAS28 were significant predictors of severe subclinical atherosclerosis (odds ratios of 1.08, 2.77, and 2.52, respectively).

**Conclusions:**

Significant associations of RA-related inflammation with lipid profiles and IR indicate the involvement of RA in atherosclerosis pathogenesis. Biologic therapies were associated with IR reduction without change in atherogenic index, but their beneficial effects on atherosclerosis reduction need to be verified in the future.

## Introduction

Rheumatoid arthritis (RA) is a chronic inflammatory articular disease [1,2] that is complicated by accelerated atherosclerosis and subsequently leads to adverse cardiovascular (CV) events [3,4]. Epidemiological studies have disclosed an increased risk of premature atherosclerosis and an increased mortality due to CV events in patients with RA [5-7].

Atherosclerosis-associated CV diseases (CVDs) are attributable to the traditional risk factors, including hypertension, dyslipidemia, diabetes mellitus (DM), and smoking in the general population [8,9]. A recent meta-analysis of traditional risk factors for CVD in patients with RA indicated an important role of low levels of high-density lipoprotein cholesterol (HDL-C) and an increased frequency of DM [10]. A nationwide cohort study demonstrates that RA is associated with the same risk of myocardial infarction as DM [11].

RA-related inflammation that is responsible for synovial lesions may be implicated in the development of accelerated atherosclerosis, leading to increased risk of CVD [12,13]. Furthermore, the magnitude and chronicity of inflammation strongly correlated with the emergence of premature atherosclerosis in RA [3,6,12,14]. The positivity of rheumatoid factor (RF) or anti-cyclic citrullinated peptide (anti-CCP) antibodies or both appears to be associated with high prevalence of subclinical atherosclerosis in RA [15]. In addition, the presence of HLA-DRB1*04 shared epitope alleles and tumor necrosis factor (TNF)A-308 (rs1800629) gene polymorphism is associated with a higher risk of CVD in patients with RA [16,17].

Recent clinical studies identified elevated levels of pro-inflammatory cytokines, including TNF-α and interleukin-6 (IL-6), as independent variables in association with arthrosclerosis in rheumatic patients and the general population [13,14,18]. TNF-α causes deterioration of the lipid profile and promotes insulin resistance (IR), both of which are traditional risk factors for atheroscerlosis [14,18]. Therefore, TNF-α inhibitors can induce favorable changes in lipid profiles with alteration of HDL composition [19]. Although previous studies failed to show that anti-TNF-α therapy could lower the risk of CVD [20,21], accumulating evidence suggests that TNF-α inhibitors can reduce the risk of future CV events in RA [22]. Besides the improvement of endothelial function [23], the possible mechanisms include a decrease of RA-associated inflammation, improvement of lipid profile [19], and the reduction of IR [24]. IL-6, a pro-inflammatory cytokine, may play a central role in decreasing total cholesterol (TC) levels and may also contribute to an increased IR in RA [25,26]. Tocilizumab, a humanized monoclonal antibody against IL-6 receptor (IL-6R), is effective in the treatment of RA [27,28]. Tocilizumab induced elevation of low-density lipoprotein cholesterol (LDL-C) but altered HDL particles toward an anti-inflammatory composition in RA [29]. These observations indicate that the reduction of RA-related inflammation and modulation of atherosclerosis-associated cytokines could be a potential strategy for the prevention of atherosclerosis in patients with RA.

Ultrasonography (US) of the carotid artery provides a non-invasive method for identifying atherosclerotic plaques, which reflect severe subclinical atherosclerosis and may predict the emergence of adverse CV events [30-33]. Common carotid artery intima-media thickness (ccIMT) measurements were shown to reflect the extent of coronary atherosclerosis [30,31]. Previous studies also showed that an increased ccIMT and evidence of plaques could predict the emergence of CVD in patients with RA [31,32]. Therefore, increased ccIMT or carotid plaques or both could be used as the gold standard for identification of severe subclinical atherosclerosis and patients at high risk of CVD [31-33].

The main objectives of this study were (1) to evaluate the associations of RA-related inflammation or RF/anti-CCP positivity with serum levels of lipid profile, atherogenic index (AI), modified Framingham CV risk score (mFRS), IR, and pro-inflammatory cytokines; (2) to investigate the effects of biologic therapy on serum levels of lipid profiles, AI, mFRS, and IR in patients with RA; and (3) to examine the potential predictive factors and their optimal cutoff value for the presence of severe subclinical atherosclerosis.

## Methods

### Patients

In total, 92 consecutive biologic-naïve patients who fulfilled the 2010 revised criteria of the American College of Rheumatology for RA [34] were enrolled. Each of them had received traditional synthetic disease-modifying anti-rheumatic drugs (tsDMARDs), including methotrexate (MTX). Seventy-two patients started anti-TNF-α or anti-IL-6R therapy in combination with a stable dose of MTX 7.5 to 15 mg weekly in accordance with the guidelines of the British Society for Rheumatology [35], whereas the other 20 patients continued with MTX therapy and other tsDMARDs (as the disease control). Patients with a recent history (within 1 year) of coronary heart disease or cerebral ischemic stroke were excluded from this study. Thirty-two patients received adalimumab at a dose of 40 mg every other week, 16 patients received etanercept at a dose of 25 mg twice weekly, and 24 patients received tocilizumab at a dose of 4 mg/kg once monthly during the first 3 months and then 8 mg/kg once monthly afterward, with concomitant MTX at a stable dose of 7.5 to 15 mg weekly. The doses of tsDMARDs as well as oral corticosteroids remained unchanged and the use of intra-articular or parenteral corticosteroids was prohibited during the period of investigation. Disease activity was assessed by the 28-joint disease activity score (DAS28) at baseline and after 6 months of biologic or tsDMARD-alone therapy, respectively [36]. The therapeutic response was evaluated after 6 months of therapy by using the European League Against Rheumatism (EULAR) response criteria [37]. We defined EULAR responders as patients with good and moderate EULAR therapeutic responses. The Institutional Review Board of Taichung Veterans General Hospital approved this study (CE12274), and the written consent of all participants was obtained in accordance with the Declaration of Helsinki.

#### Measurements of serum levels of lipid profiles and atherogenic index

All measurements were performed after an overnight fasting of 12 hours at baseline and after 6 months of biologic or tsDMARD-alone therapy, respectively. Serum levels of TC, triglyceride (TG), HDL-C, and LDL-C were measured by using enzymatic methods by a chemistry analyzer (Hitachi 7600; Hitachi, Tokyo, Japan) in accordance with the instructions of the manufacturer. The AI, which corresponds to the ratio of TC to HDL-C, was calculated.

#### Measurements of the Framingham risk score and modified Framingham risk score

FRS was determined on the basis of the demographic data and traditional risk factors [38]. The FRS estimates the 10-year risk of adverse CV events, including heart attack, stroke, or other occlusive arterial disease. Considering additional risks for patients with RA, we adopt a modified FRS (mFRS) by the application of a multiplier factor of 1.5 to those with two of the following three criteria: disease duration of more than 10 years, RF or anti-CCP antibody positivity, and the presence of extra-articular manifestations [39].

#### Measurements of insulin resistance

Serum insulin levels were determined by using a commercially available assay kit (IMMULITE, I-2000; EURO/Diagnostic Products Cooperation, Gwynedd, UK). Homeostasis model assessment for IR (HOMA-IR) was calculated by using this formula: fasting plasma insulin (in micro-international units per liter) × fasting plasma glucose (in millimoles per liter)/22.5 [40].

#### Ultrasound vascular imaging of carotid artery

Ultrasound vascular imaging for carotid arteries included measurement of ccIMT and detection of focal plaques in the extracranial carotid tree. The final ccIMT was represented by the largest average ccIMT measured at the far wall of the common carotid arteries along a 10-mm section of the artery proximal to the carotid bifurcation, and the proximal 15-mm-long segment of the internal and external carotid arteries. Plaque was defined as a localized thickening of more than 1.5 mm that did not uniformly involve the whole artery according to Mannheim consensus criteria [41]. A ccIMT of more than 0.90 mm or carotid plaques (or both) is defined as the gold standard for severe subclinical atherosclerosis [42].

#### Determination of serum levels of pro-inflammatory cytokines

Serum levels of TNF-α and IL-6 were determined in 32 adalimumab-treated patients, 16 etanercept-treated patients, 14 tocilizumab-treated patients, and 10 biologic-naïve patients by using an enzyme-linked immunosorbent assay (PeproTech Inc., Rocky Hill, NJ, USA) in accordance with the instructions of the manufacturer.

#### Statistical analysis

Results are presented as the mean ± standard deviation (SD) or standard error of mean (SEM). The non-parametric Kruskal-Wallis test was used for comparisons between groups. When this test showed a significant difference, the exact *P* value was determined by using the Mann-Whitney *U* test. The independent samples *t* test was used for between-group (positivity and negativity for RF/anti-CCP antibodies) comparison of baseline levels of lipid profiles, AI, mFRS, IR, and pro-inflammatory cytokines. The non-parametric Spearman’s correlations were determined between RA disease activity (DAS28) and levels of lipid profiles, AI, mFRS, IR, or pro-inflammatory cytokines. For comparison of levels of lipid profiles, AI, mFRS, IR, and pro-inflammatory cytokines during follow-up in RA patients before and after biologic therapy, the Wilcoxon signed rank test was employed. We also constructed a logistic regression model to evaluate the contribution of traditional risk factors and RA-related risk factors to the presence of severe subclinical atherosclerosis detected by US. The optimal cutoff values of age, AI, mFRS, IR, and DAS28 for the occurrence of severe subclinical atherosclerosis was determined by using receiver operating characteristic (ROC) curve analysis. The diagnostic sensitivity and specificity were determined by using MedCalc statistical software version 9.3 (MedCalc Software, Ostend, Belgium). A probability of less than 0.05 was considered significant.

## Results

### Clinical characteristics of patients with rheumatoid arthritis

As illustrated in Table 1, the majority of patients with RA were women and all patients had active disease (DAS28 of more than 3.2) at baseline. Among these patients, 78.3% were positive for RF and 69.6% were positive for anti-CCP antibodies; 58 (63.0%) had a disease duration of at least 10 years; 13 (14.1%) patients had extra-articular manifestations, including secondary Sjögren’s syndrome in eight patients, interstitial lung disease in four, pleuritis/pericarditis in two, rheumatoid nodule in one, and rheumatoid vasculitis in one). According to the results of carotid US, 57.1% of patients with RA had severe subclinical atherosclerosis. There were no significant differences in the positive rate of RF/anti-CCP antibodies, daily dose of corticosteroids, weekly dose of MTX, or proportion of tsDMARDs used among four subgroups of RA patients at baseline.

**Table 1:** **Demographic data and laboratory findings at baseline in four subgroups of rheumatoid arthritis patients according to therapeutic agents used**

	**Adalimumab (n = 32)**	**Etanercept (n = 16)**	**Tocilizumab (n = 24)**	**Without biologic (n = 20)**
Mean age, years	53.5 ± 12.6	54.4 ± 7.8	56.8 ± 14.4	57.0 ± 11.4
Female proportion	28 (87.5%)	13 (81.3%)	20 (83.3%)	17 (85.0%)
Disease duration, years	13.4 ± 6.6	13.6 ± 9.0	13.0 ± 9.7	12.3 ± 5.8
Body mass index, kg/m^2^	24.8 ± 4.6	23.6 ± 4.5	24.5 ± 3.7	22.9 ± 2.9
RF positivity	28 (87.5%)	13 (81.3%)	17 (70.8%)	14 (70.0%)
Anti-CCP positivity	21 (65.6%)	11 (68.8%)	17 (70.8%)	15 (75.0%)
Baseline ESR, mm/1st hour	33.5 ± 23.4	39.0 ± 36.0	42.5 ± 30.0	35.8 ± 22.1
Baseline DAS28	5.46 ± 0.94	5.48 ± 0.98	5.80 ± 0.85	5.12 ± 0.59
Daily steroid dose, mg/day	6.3 ± 2.5	6.9 ± 1.9	7.4 ± 2.1	7.0 ± 2.2
DMARDs at baseline				
MTX, weekly dose, mg	12.0 ± 2.5	10.6 ± 3.7	11.5 ± 2.5	11.3 ± 2.8
Sulfasalazine	27 (84.4%)	12 (75.0%)	18 (75.0%)	17 (85.0%)
Hydroxychloroquine	24 (75.0%)	12 (75.0%)	19 (79.2%)	18 (90.0%)
Cyclosporine	6 (18.7%)	4 (25.0%)	5 (20.8%)	7 (35.0%)
Hypertension	14 (43.8%)	6 (37.5%)	8 (33.3%)	8 (40.0%)
Antihypertensive Rx	8 (25.0%)	4 (25.0%)	7 (29.2%)	6 (30.0%)
Diabetes mellitus	2 (6.2%)	1 (6.3%)	1 (4.2%)	1 (5.0%)
Hypoglycemic Rx	1 (3.1%)	1 (6.3%)	0 (0.0%)	0 (0.0%)
Current smoker	3 (9.4%)	2 (16.6%)	3 (12.5%)	3 (15.0%)

Data are presented as mean ± standard deviation or as number (percentage). anti-CCP, anti-cyclic citrullinated peptide (antibodies); DAS28, disease activity score for 28 joints; DMARDs, disease-modifying anti-rheumatic drugs; ESR, erythrocyte sedimentation rate; MTX, methotrexate; RF, rheumatoid factor; Rx, treatment.

After 6 months of biologic or tsDMARD-alone therapy, 25 (78.1%) adalimumab-treated patients, 13 (81.3%) etanercept-treated patients, and 20 (83.3%) tocilizumab-treated patients were EULAR responders. However, only 50% of biologics-naïve patients were EULAR responders. The mean DAS28 changes (± SDs) were 2.00 ± 0.88 in adalimumab-treated patients, 1.86 ± 0.92 in etanercept-treated patients, and 2.13 ± 0.99 in tocilizumab-treated patients, while a significantly lower change in DAS28 (1.05 ± 0.56) was observed in biologics-naïve patients (*P* <0.001, *P* <0.01, and *P* <0.001, respectively).

### Associations of rheumatoid factor/anti-CCP antibody positivity with lipid profiles, atherogenic index, modified Framingham risk score, insulin resistance, and pro-inflammatory cytokines at baseline

**Figure 1:**
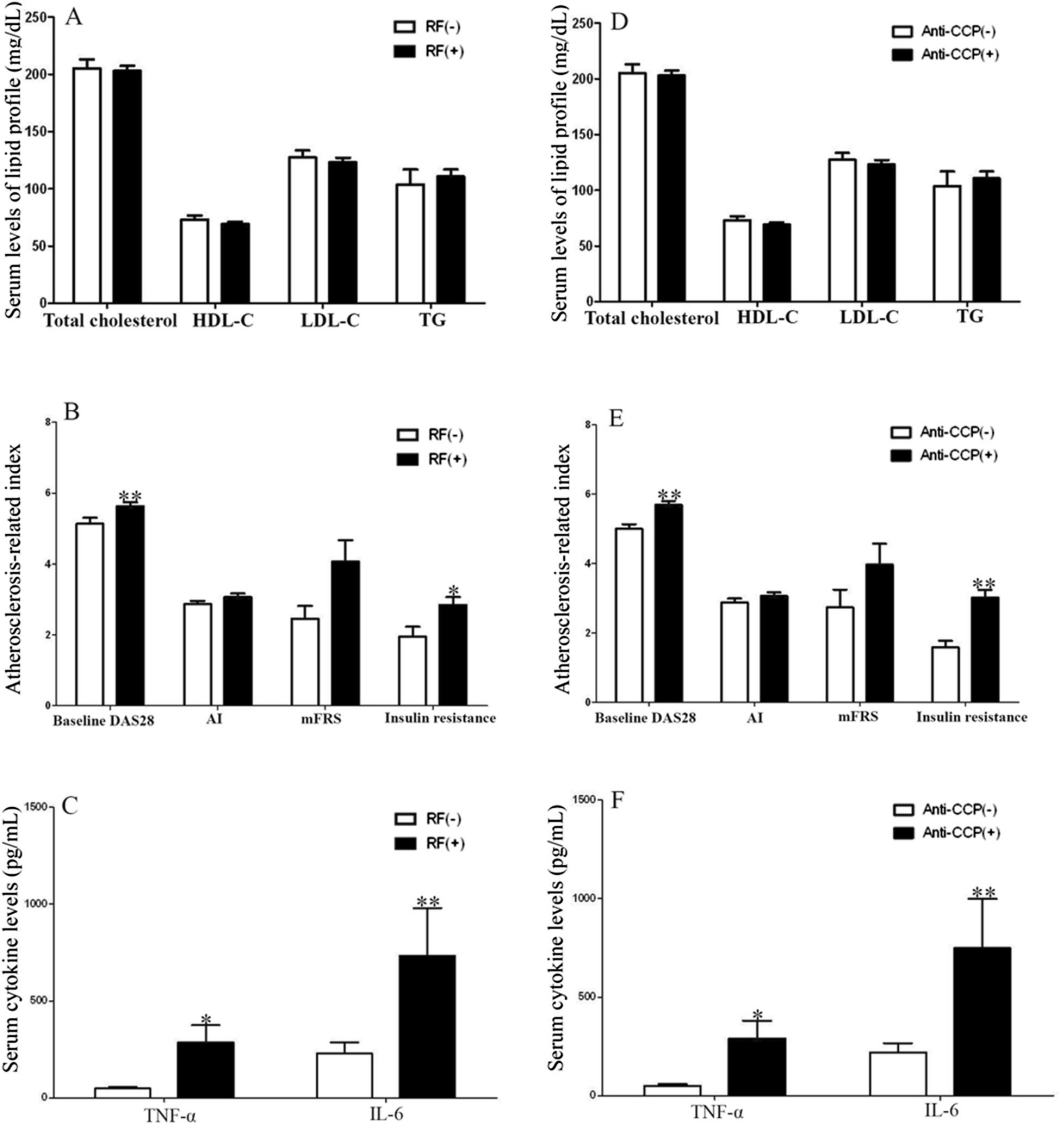
**The associations of RF/anti-CCP antibody positivity with lipid profiles. (A)** Comparisons of serum levels of lipid profile between RA patients with positive rheumatoid factor (RF) [RF(+)] and negative RF [RF(−)]. **(B)** Comparisons of baseline DAS28, atherogenic index (AI), modified Framingham CV risk scores (mFRS), insulin resistance between RA patients with positive RF and negative RF. **(C)** Comparisons of proinflammatory cytokines between RA patients with positive RF and negative RF. **(D)** Comparisons of serum levels of lipid profile between RA patients with positive anti-CCP antibodies [anti-CCP (+)] and negative anti-CCP antibodies [anti-CCP (−)]. **(E)** Comparisons of baseline DAS28, atherogenic index (AI), modified Framingham CV risk scores (mFRS), insulin resistance between RA patients with positive anti-CCP antibodies and negative anti-CCP antibodies. **(F)** Comparisons of proinflammatory cytokines between RA patients with positive anti-CCP antibodies and negative anti-CCP antibodies.

Compared with patients with negative RF, those with positive RF had significantly higher baseline DAS28 (mean ± SEM, 5.63 ± 0.11 versus 5.13 ± 0.17, *P* <0.01), IR (2.85 ± 0.22 versus 1.95 ± 0.28, *P* <0.05), TNF-α levels (285.61 ± 87.78 pg/mL versus 48.19 ± 8.03 pg/mL, *P* <0.05), and IL-6 levels (734.16 ± 245.38 pg/mL versus 230.28 ± 54.94 pg/mL, *P* <0.01) (Figure 1A-C). Similarly, anti-CCP-positive patients had significantly higher values in these parameters compared with anti-CCP-negative patients: baseline DAS28 (5.69 ± 0.11 versus 5.00 ± 0.13, *P* <0.01), IR (3.02 ± 0.22 versus 1.58 ± 0.19, *P* <0.01), TNF-α levels (289.74 ± 89.45 pg/mL versus 49.58 ± 8.23 pg/mL, *P* <0.05), and IL-6 levels (748.48 ± 250.21 versus 220.63 ± 45.34, *P* <0.01) (Figure 1D-F). However, there were no significant differences in serum levels of lipid profile, AI, or mFRS between seropositive patients and seronegative patients.

### Association of rheumatoid arthritis DAS28 with lipid profiles, atherogenic index, modified Framingham risk score, insulin resistance, DAS28, and pro-inflammatory cytokines at baseline

**Figure 2:**
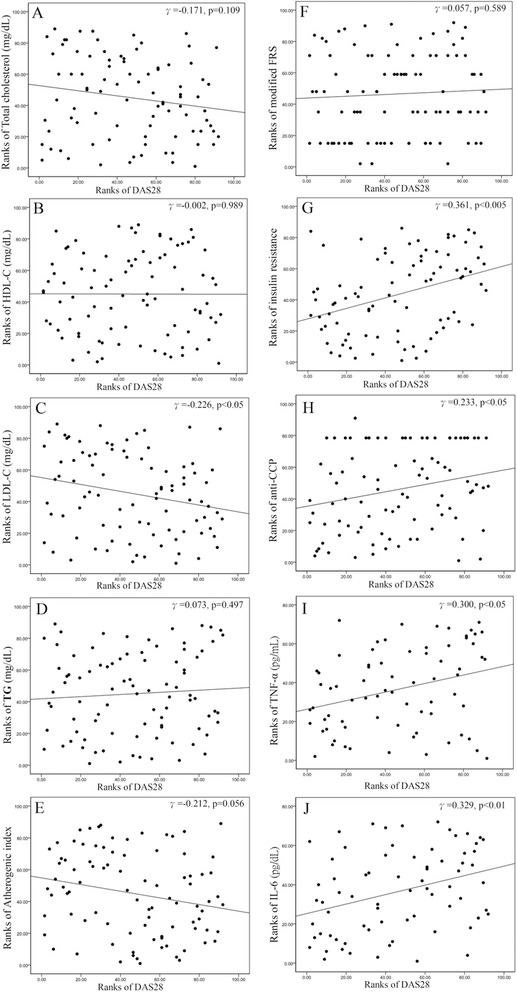
**The association of disease activity (DAS28) with lipid profiles, AI, mFRS, IR, and proinflammatory cytokines. (A)** The correlation between DAS28 and serum levels of total cholesterol. **(B)** The correlation between DAS28 and serum levels of HDL-C. **(C)** The correlation between DAS28 and serum levels of low-density LDL-C. **(D)** The correlation between DAS28 and serum levels of TG. **(E)** The correlation between DAS28 and atherogenic index (AI). **(F)** The correlation between DAS28 and modified Framingham CV risk scores (FRS). **(G)** The correlation between DAS28 and insulin resistance (IR). **(H)** The correlation between DAS28 and anti-CCP levels. **(I)** The correlation between DAS28 and serum levels of TNF-α. **(J)** The correlation between DAS28 and serum levels of IL-6.

As shown in Figure 2, there was an inverse correlation between DAS28 and LDL-C levels and a positive correlation between DAS28 and IR, anti-CCP level, or levels of cytokines, including TNF-α and IL-6. However, there was no significant correlation between DAS28 and AI or mFRS.

### Correlation between cytokine levels and lipid profiles, atherogenic index, modified Framingham risk score, or insulin resistance at baseline

There was an inverse correlation between IL-6 levels and LDL-C levels (r = −0.269, *P* <0.05) and a positive correlation between IL-6 levels and IR (r = 0.326, *P* <0.01). There was a positive correlation between TNF-α levels and IR (r = 0.416, *P* <0.005). However, there was no significant correlation between cytokine levels and lipid profiles or mFRS.

### Change in lipid profiles, atherogenic index, modified Framingham risk score, and insulin resistance after therapy with or without biologics

**Figure 3:**
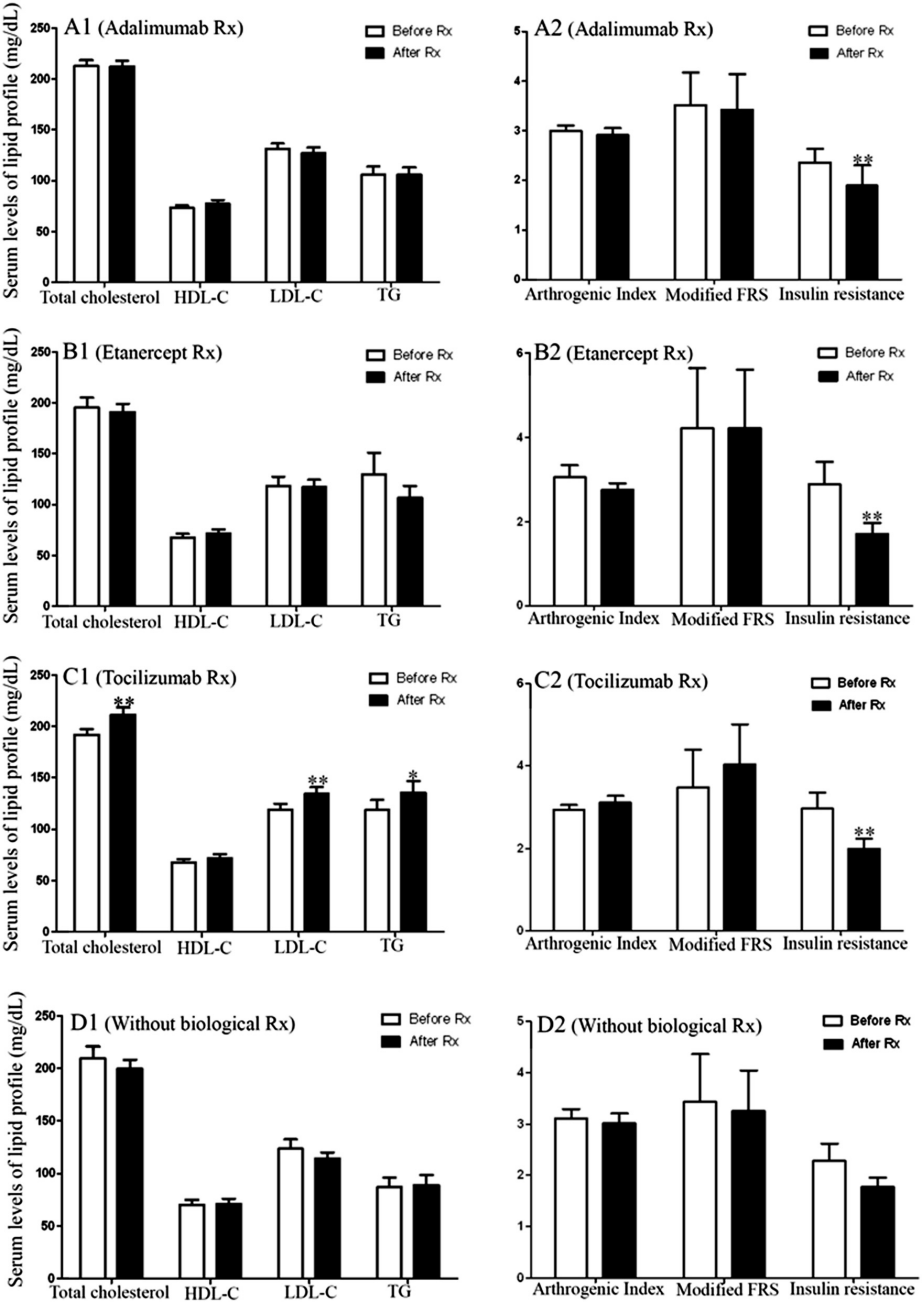
**Change in lipid profiles, AI, mFRS, and IR after therapy with or without biologics. (A1)** The changes in serum levels of lipid profile after 6 months of therapy in adalimumab-treated patients. **(A2)** The changes in AI, mFRS, and IR after 6 months of therapy in adalimumab-treated patients. **(B1)** The changes in serum levels of lipid profile after 6 months of therapy in etanercept-treated patients. **(B2)** The changes in AI, mFRS, and IR after 6 months of therapy in etanercept-treated patients. **(C1)** The changes in serum levels of lipid profile after 6 months of therapy in tocilizumab-treated patients. **(C2)** The changes in AI, mFRS, and IR after 6 months of therapy in tocilizumab-treated patients. **(D1)** The changes in serum levels of lipid profile after 6 months treatment in biologic-naïve patients. **(D2)** The changes in AI, mFRS, and IR after 6 months treatment in biologic-naïve patients.

Serum HDL-C levels significantly increased (mean ± SEM, 71.29 ± 2.24 mg/dL versus 75.40 ± 2.88 mg/mL, *P* <0.05) in patients receiving 6 months of anti-TNF-α therapy. As shown in Figure 3 and Table 2, there was no significant change in serum levels of lipid profiles, AI, or mFRS in patients receiving 6 months of adalimumab or etanercept. Among tocilizumab-treated patients, levels of TC, LDL-C, and TG significantly increased without significant changes in HDL-C levels or AI. It is interesting that IR significantly decreased after 6 months of biologic therapy, including adalimumab, etanercept, or tocilizumab. However, there was no significant change in levels of lipid profile, AI, mFRS, or IR in those not treated with biologic therapy.

**Table 2:** **Change in lipid profiles, AI, mFRS, and IR in patients receiving biologic or biologic-naïve therapy**

	**Adalimumab (n = 32)**	**Etanercept (n = 16)**	**Tocilizumab (n = 24)**	**Without biologic (n = 20)**
Total cholesterol, mg/dL				
Baseline	212.3 ± 5.8	195.3 ± 9.8	191.8 ± 5.3	209.6 ± 11.2
Week 24	212.1 ± 5.6	191.0 ± 7.9	211.5 ± 6.8*	199.5 ± 8.6
HDL-C, mg/dL				
Baseline	73.0 ± 2.7	67.5 ± 4.1	67.7 ± 3.3	70.4 ± 4.4
Week 24	77.1 ± 3.8	71.7 ± 4.0	72.1 ± 4.0	70.8 ± 5.2
LDL-C, mg/dL				
Baseline	131.3 ± 5.3	118.4 ± 8.9	119.2 ± 5.5	123.4 ± 9.1
Week 24	127.0 ± 5.6	117.0 ± 7.4	134.3 ± 6.5*	114.2 ± 6.0
Triglyceride, mg/dL				
Baseline	105.6 ± 8.3	129.8 ± 21.1	118.8 ± 9.8	86.9 ± 9.3
Week 24	105.7 ± 7.1	106.4 ± 12.0	135.5 ± 11.4*	88.9 ± 9.6
Atherogenic index				
Baseline	3.00 ± 0.11	3.05 ± 0.29	2.94 ± 0.12	3.11 ± 0.18
Week 24	2.91 ± 0.14	2.75 ± 0.16	3.11 ± 0.16	3.01 ± 0.20
Modified FRS				
Baseline	3.52 ± 0.65	4.22 ± 1.44	3.48 ± 0.91	3.43 ± 0.93
Week 24	3.42 ± 0.72	4.22 ± 1.40	4.04 ± 0.97*	3.25 ± 0.79
Insulin resistance				
Baseline	2.75 ± 0.29	2.90 ± 0.53	2.97 ± 0.38	2.28 ± 0.34
Week 24	1.76 ± 0.39*	1.71 ± 0.26**	1.99 ± 0.25**	1.77 ± 0.18

Data are presented as mean ± standard error of mean. *P <0.05, **P <0.005, versus before treatment, determined by Wilcoxon signed rank test. FRS, Framingham risk score; HDL-C, high-density lipoprotein cholesterol; LDL-C, low-density lipoprotein cholesterol.

### Logistic regression analysis

As illustrated in Table 3, univariate regression analysis demonstrated that age, AI, mFRS, IR, disease duration of more than 10 years, and DAS28 were identified as potential predictors of severe subclinical atherosclerosis (odds ratio, 1.09, *P* <0.001; 2.12, *P* <0.05; 1.20, *P* <0.05; 2.43, *P* <0.001; 3.33, *P* <0.01; and 2.94, *P* <0.005, respectively). To establish the best model to predict subclinical atherosclerosis, multivariate regression analysis was performed by choosing the variables that were significant by univariate regression analysis. Age, IR, and DAS28 were demonstrated as significant predictors of severe subclinical atherosclerosis (odds ratio, 1.08, *P* <0.01; 2.77, *P* <0.005; and 2.52, *P* <0.05; respectively).

**Table 3:** **Univariate and multivariate association of traditionalcardiovascular risk factors and RA-rated factors with severe subclinical atherosclerosis**

**Risk factors (univariate)**	**Odds ratio**	**95% confidence interval**	***P*** **value**
Age	1.09	1.04-1.14	0.000
Sex (female)	0.89	0.18-4.39	0.883
Smoking status	3.87	0.79-19.06	0.096
Hypertension Rx.	1.35	0.55-3.32	0.517
Body mass index	0.95	0.86-1.05	0.328
Total cholesterol	1.00	0.98-1.01	0.491
HDL-C	1.01	0.98-1.04	0.467
LDL-C	1.00	0.98-1.01	0.511
Total triglyceride	1.00	0.99-1.02	0.640
Artherogenic index	2.12	1.05-4.25	0.035
Modified FRS	1.20	1.01-1.42	0.033
Insulin resistance	2.43	1.50-3.93	0.000
Disease duration >10 years	3.33	1.36-8.15	0.008
RF positivity	1.41	0.44-4.44	0.562
Anti-CCP positivity	1.44	0.89-2.35	0.142
TNF-α levels	1.00	0.99-1.00	0.743
IL-6 levels	1.00	1.00-1.00	0.196
DAS28	2.94	1.58-5.49	0.001
**Risk factors (multivariate)**	**Odds ratio**	**95% confidence interval**	***P*** **value**
Age	1.08	1.02-1.15	0.007
Artherogenic index	1.31	0.98-1.76	0.073
Modified FRS	1.17	0.97-1.40	0.093
Insulin resistance	2.77	1.40-5.46	0.003
Disease duration >10 years	5.89	0.93-37.16	0.059
DAS28	2.52	1.00-6.35	0.049

Anti-CCP, anti-cyclic citrullinated peptide (antibodies); DAS28, disease activity score for 28-joints; FRS, Framingham risk score; HDL-C, high-density lipoprotein cholesterol; IL-6, interleukin-6; LDL-C, low-density lipoprotein cholesterol; RF, rheumatoid factor; Rx, treatment; TNF-α, tumor necrosis factor-alpha.

### Determination of the optimal cutoff values of predictive variables by receiver operating characteristic analysis

The optimal cutoff value of age for predicting the occurrence of severe subclinical atherosclerosis was 59 years (area under ROC curve [AUC] of 0.784, sensitivity of 63.5%, and specificity of 84.6%, *P* <0.001), the optimal cutoff value of AI was 3.02 (AUC of 0.654, sensitivity of 55.8%, and specificity of 71.8%, *P* <0.01), the optimal cutoff value of mFRS was 2.0 (AUC of 0.701, sensitivity of 59.6%, and specificity of 76.9%, *P* <0.001), the optimal cutoff value of IR was 1.27 (AUC of 0.791, sensitivity of 94.2%, and specificity of 53.9%, *P* <0.001), and the optimal cutoff value of DAS28 was 5.28 (AUC of 0.741, sensitivity of 67.3%, and specificity of 76.9%, *P* <0.001).

## Discussion

In the present study, we demonstrated an inverse correlation between RA-related inflammation (DAS28) and serum LDL-C levels and a positive correlation between DAS28 and IR as well as levels of cytokines, including TNF-α and IL-6, at baseline. Moreover, patients who were seropositive for RF or anti-CCP antibodies had significantly higher DAS28, IR, TNF-α levels, and IL-6 levels compared with seronegative patients. After 6 months of biologic therapy, serum HDL-C levels significantly increased in patients receiving anti-TNF-α therapy, and levels of TC, LDL-C, and TG significantly increased in tocilizumab-treated patients. IR significantly decreased after 6 months of therapy with TNF-α inhibitors or IL-6R inhibitor, but AI did not show significant change. There was no significant change in levels of lipid profiles, AI, mFRS, or IR in patients who did not receive biologic therapy. In addition, multivariate regression analysis revealed that age, IR, and DAS28 were potential predictors of severe subclinical atherosclerosis.

Dyslipidemia is a well-established traditional risk factor for atherosclerosis [4,9,10,43]. Although the differences in the lipid profile between RA patients and the general population remain to be clarified, RA-related inflammation may be responsible for the change in lipid profiles [13-15,44]. Recent studies demonstrated that female patients with RA had significantly lower LDL-C levels than women in the general population [45,46]. Consistent with the findings of a previous study [44], our results showed an inverse correlation between RA-related inflammation (DAS28) and LDL-C levels in patients with RA. The lower LDL-C levels, in conjunction with elevated risk of CVDs in RA patients compared with the general population [5-7,46], support the hypothesis of a lipid paradox in this disease [44]. When compared with seronegative patients for RF or anti-CCP antibodies, our seropositive patients had significantly higher IR, consistent with a recent report that seropositivity is associated with IR in patients with inflammatory polyarthritis [47]. In agreement with a previous study that the presence of anti-CCP antibodies appears to be associated with a high prevalence of subclinical atherosclerosis in RA [15], we demonstrated that patients with anti-CCP positivity had higher IR and DAS28, which have been implicated in the occurrence of atherosclerosis.

Accumulating evidence indicates that the accelerated atherosclerosis in RA cannot be explained by traditional risk factors alone [44]. Our results showed a positive correlation between RA disease activity (DAS28) and IR, which is an independent risk factor for atherosclerotic CVD [48]; these findings suggest that RA-related inflammation, as reflected by DAS28, is important in the emergence of IR. Our data also were consistent with the findings that patients with high-grade inflammation were more likely to have a high IR than those with low-grade inflammation [49]. In addition, pro-inflammatory cytokines, including TNF-α and IL-6, which are involved in RA pathogenesis, play a critical role in atherosclerosis in patients with RA [14,18]. In the present study, we demonstrated that levels of TNF-α and IL-6 were positively correlated with both DAS28 and IR in patients with RA. Our results were consistent with those of previous studies showing that TNF-α promotes IR [14,18] and that IL-6 may contribute to an elevated IR [25,26]. Furthermore, our observations support the conclusion that RA-related inflammation and pro-inflammatory cytokines play an increasingly important role in the pathogenesis of IR in patients with RA [50].

During a longitudinal follow-up of patients with RA, we found that HDL-C levels were significantly increased in patients receiving 6 months of anti-TNF-α therapy, consistent with the results of previous studies [19,51,52]. In agreement with a systemic review with meta-analysis [53], our study showed no significant changes in LDL-C levels or AI in patients receiving 6 months of anti-TNF-α therapy. The findings of increases in levels of TC, LDL-C, and TG without apparent change in AI in our patients under anti-IL-6R therapies were consistent with previous reports [28,29,54,55]. These observations suggest that AI is less susceptible to the fluctuation of disease activity, making it more appropriate for predicting CVD risk in RA patients than individual lipid levels. Whether the changes in lipid profiles after biologic therapy contribute to CVD risk remains unclear.

It is interesting that there was a significant decrease in IR in patients receiving 6 months of anti-TNF-α therapy but that there was no significant change in IR in those receiving biologic-naïve therapies. Our results were similar to the findings of a study showing that knockout mice lacking TNF-α expression demonstrated improved IR [56], and they were consistent with recent studies showing beneficial effects of anti-TNF-α therapy on IR [24,52]. However, Rosenvinge *et al*. reported no significant change in IR from baseline in nine RA patients after 2 months of adalimumab therapy [57]. The discrepancy may be caused by the small number of subjects and a short-term therapy that was not long enough to detect treatment-related changes in IR. Similarly, we demonstrated a significant decrease in IR in patients receiving 6 months of anti-IL-6R (tocilizumab) therapy. Our results were consistent with a recent study that tocilizumab therapy decreases IR in patients with RA [58]. These observations imply that biologic therapies that block TNF-α or IL-6 reduce IR, which is critical in the development of atherosclerosis in patients with RA.

To identify potential predictors for the occurrence of severe subclinical atherosclerosis [42], a logistic regression analysis was performed. Univariate analysis demonstrated that age, AI, mFRS, IR, disease duration of more than 10 years, and DAS28 were significant predictors of severe subclinical atherosclerosis. Our results supported previous studies revealing that age, AI, and FRS at baseline were associated with the occurrence of subclinical atherosclerosis in patients with RA [59,60]. Our data were also similar to another study reporting that IR was an individual predictor for identifying subjects with carotid plaque [61]. Disease duration of more than 10 years was a significant predictor of subclinical atherosclerosis in this study resonated with previous findings that a higher ccIMT was observed in RA patients with longer disease duration [62], and supported the claim that RA patients with disease duration of more than 10 years would need to undergo carotid US [63]. DAS28 was also a significant predictor of subclinical atherosclerosis, consistent with the findings of previous studies showing that RA-related inflammation is implicated in the development of atherosclerosis [12,13]. Similar to the results of other studies [15,64], we demonstrated a trend of an association of anti-CCP positivity with the occurrence of subclinical atherosclerosis (p=0.142). However, our findings should be verified by further extensive studies because of the use of a large number of variates in this small sample of patients.

Selection of the optimal cutoff value of age, AI, mFRS, IR, and DAS28 at baseline for predicting severe subclinical atherosclerosis may have clinical implications. Using ROC analysis, we demonstrated that patients with age above 59 years, AI above 3.02, mFRS above 2.0, IR above 1.27, and DAS28 above 5.28 might have a high probability of developing severe subclinical atherosclerosis. However, our results are preliminary; hence, the use of these cutoff values in clinical practice cannot be recommended until their external validity has been confirmed.

Some limitations in this study should be addressed. This is a preliminary study that enrolled a limited number of active RA patients who were followed up for 6 months. Actual CV events and mortality were not available, and positive results of carotid US were used as a surrogate marker of severe subclinical atherosclerosis. In consideration of the effects of corticosteroids and multiple DMARDs on levels of lipid profile, we did not analyze the differences in baseline levels of lipid profiles between RA patients and healthy subjects. However, recent studies and a meta-analysis have demonstrated the differences in lipid profiles between RA patients and a healthy population [10,45,46]. In addition, none of the enrolled patents in our study was in an early stage of RA and this may limit the generalizability of these results to the whole population. A long-term study enrolling larger groups of RA patients, including an early RA population, who receive biologic therapy and tsDMARDs alone is required to confirm these data. Because adipokines are of main relevance in the development of the metabolic syndrome frequently observed in patients with RA, the investigation of the effects of biologic therapy on adipokines [65] would be needed. Finally, qualitative changes in lipid profile such as HDL-C subfractions [66] and small dense LDL-C particles were not addressed.

## Conclusions

Our results show significant associations of RA-related inflammation with LDL-C levels and IR. Patients with seropositive RA had significantly higher DAS28, IR, and levels of pro-inflammatory cytokines at baseline when compared with seronegative patients. Biologic therapies are associated with a significant increase in HDL-C levels (TNF-α inhibitors) or levels of TC, LDL-C, and TG (IL-6R inhibitor) without apparent change in AI. Biologic therapies could also improve insulin sensitivity. Age, AI, mFRS, IR, long disease duration, and DAS28 could predict the emergence of subclinical atherosclerosis in patients with RA. Early identification of traditional risk factors, tight control of RA-related inflammation with biological therapy, and ongoing monitoring of CVD risk factors are mandatory for slowing the progression of atherosclerosis in patients with RA.

## Abbreviations

AI: atherogenic index
anti-CCP: anti-cyclic citrullinated peptide
AUC: area under receiver operating characteristic curve
ccIMT: common carotid artery intima-media thickness
CV: cardiovascular
CVD: cardiovascular disease
DAS28: 28-joint disease activity score
DM: diabetes mellitus
DMARD: disease-modifying anti-rheumatic drug
EULAR: European League Against Rheumatism
FRS: Framingham risk score
HDL-C: high-density lipoprotein cholesterol
IL-6: interleukin-6
IL-6R: interleukin-6 receptor
IR: insulin resistance
LDL-C: low-density lipoprotein cholesterol
mFRS: modified Framingham risk score
MTX: methotrexate
RA: rheumatoid arthritis
RF: rheumatoid factor
ROC: receiver operating characteristic
SD: standard deviation
SEM: standard error of the mean
TC: total cholesterol
TG: triglyceride
TNF: tumor necrosis factor
tsDMARD: traditional synthetic disease-modifying anti-rheumatic drug
US: ultrasonography
